# Random Access Addressing of MEMS Electrostatic Shutter Array for Multi-Object Astronomical Spectroscopy

**DOI:** 10.3390/mi11080782

**Published:** 2020-08-17

**Authors:** Xufeng Liu, Takuya Takahashi, Masahiro Konishi, Kentaro Motohara, Hiroshi Toshiyoshi

**Affiliations:** 1 Institute of Industrial Science, The University of Tokyo, Tokyo 153-8505, Japan; lxfeng@iis.u-tokyo.ac.jp (X.L.); takuya@iis.u-tokyo.ac.jp (T.T.); 2 Institute of Astronomy, School of Science, The University of Tokyo, Tokyo 181-0015, Japan; konishi@ioa.s.u-tokyo.ac.jp; 3 National Astronomical Observatory of Japan, Tokyo 181-8588, Japan; kmotohara@ioa.s.u-tokyo.ac.jp

**Keywords:** electrostatic, actuator, multi-object spectroscopy, random access addressing

## Abstract

An extended version of cross-bar type addressing technique is developed for three-port electrostatic micro shutters arranged in an arrayed format. A microelectromechanical systems (MEMS) shutter blade suspended by a pair of torsion beams works as a movable electrode that is either attracted upwards to the cover plate to close the aperture or retracted downwards into the through-hole to open it. Tri-state positioning of the shutter—i.e., open, rest, and close—is controlled by the hysteresis loop of the electrostatic pull-in and release behavior using the combination of the voltages applied to the shutter, the cover, and the substrate. Random access addressing of the shutters is demonstrated by a control system composed of MATLAB-coded Arduino electronics. The shutter array developed in this work is for a sub-cluster of a reconfigurable shutter array under development for a multi-object galactic astronomy.

## 1. Introduction

**Figure 1:**
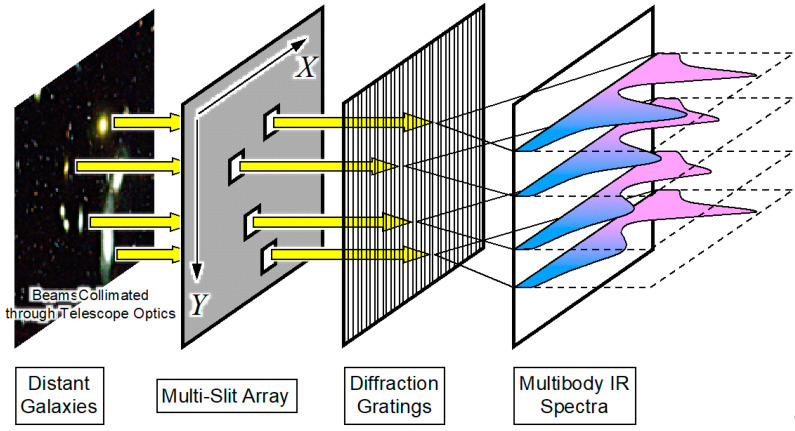
Schematic illustration of multi-object spectroscopy (MOS) for deep galactic astronomy. The starlights from distant galaxies are spatially filtered by the multi-slit array plate, and guided to the gratings to project multiple spectra on the infrared imaging device for simultaneous spectroscopy.

As an endeavor to understand the history of the universe, three dimensional maps of galaxies are being constructed using their distances estimated by the Doppler redshifts of emission lines. Multi-object spectrograph (MOS) is a powerful tool for such galactic astronomy because of its parallelism of spectroscopy analysis. Conventional MOS uses a metallic masking plate with multiple slit holes whose positions are made to match with the constellation patterns of target astronomical bodies; MOSs of this type have been implemented in the Faint Object Camera and Spectrograph (FOCAS) of the Subaru Telescope [1], DEep Imaging Multi-Object Spectrograph (DEIMOS) of the Keck II Telescope [2], and VIsible Multi-Object Spectrograph (VIMOS) of the Very Large Telescope (VLT) [3]. The general concept of MOS is schematically shown in Figure 1; spatially filtered starlights are guided into the gratings, and their spectra are projected onto a cryostat imager. Optical fibers can also be used as a spatial aperture to selectively guide the lights from the target galaxies to the spectrometer; such fiber-feed type MOSs are used in the Sloan Digital Sky Survey (SDSS) [4], 2-degree Field (2dF) MOS of the Anglo-Australian Telescope [5], the Fiber Multi-Object Spectrograph (FMOS) of the Subaru Telescope [6], Large Sky Area Multi-Object Fiber Spectroscopic Telescope (LAMOST) [7], and in the UK Schmidt Telescope (UKST) [8,9].

Because of the fixed patterns of the slit holes or the optical fibers, however, the conventional MOS operation urges the astronomers to replace the filter optics every time the target galaxies are changed. For visible wavelength spectroscope, slit masks can be easily loaded and unloaded because the mask plates are usually stored in a room-temperature environment in the spectrograph instrument housing. When more distant galaxies are observed, on the other hand, the emitted light is red-shifted into the near infrared range. In such a wavelength range, the thermal emission from the slit masks becomes major contamination to the spectroscopy, and thus the optics must be kept in a cryogenic temperature in a condensation-free vacuum environment, which makes it difficult to frequently change the masks. For instance, the multi-object infrared camera and spectrograph (MOIRCS) system on Subaru [10] utilizes a cryogenic robotic mechanism to change the slit masks; loading a new set of masks takes more than a week to wait for the mask storage warm up to room temperature and cool down again to the cryogenic temperature for operation. As a solution to enhance the throughput, reconfigurable MOS slits have been developed by using the precision mechanics. For instance, multiple rows of mechanically movable metallic bars are placed on the focal plane of the telescope, and the slit position and the opening gap are mechanically adjusted by sliding the bars [11]; nevertheless, it requires a bulky complicated mechanism that should be compatible with the cryogenic and vacuum environment.

Microelectromechanical systems (MEMS) technology is therefore expected to be an enabling solution to construct an agile and compact reconfigurable multi slit array for a MOS in a remote unmanned observatory, and several possible configurations have been proposed by Riesenberg and Wuttig [12]. As a most advanced example, NASA Goddard Space Flight Center (GSFC) has developed a micro shutter array (MSA) for the James Web Space Telescope (JSWT), in which a matrix of micromechanical shutters coated with a thin-film magnetic material is used as reconfigurable apertures to select multiple objects at a time [13]. As an addressing mechanism, they used a combination of magnetic actuation and electrostatic latch; a permanent magnet is spatially scanned beneath the micro shutter array to choose a shutter column to open, and then individual shutter elements are latched by the electrostatic attractive force induced by the cross-bar like electrical addressing lines on the shutter substrate. Operation at a cryostat temperature has been tested [14], and a flight model of MSA has been developed along with its control electronics [15].

The research groups at the University of Tokyo, on the other hand, are constructing a new ground-based infrared telescope with a reflector of 6.5 m in diameter at the Tokyo Atacama Observatory (TAO) on a 5,640-m-high mountain in Chile [16], for which a MEMS type MOS shutter is development [17,18]. Owing to the transmissive optics design of the spectroscope, this work adopted a multi-slit device similar to the MSA for the JSWT [13] rather than a reflective optics using micro mirror array (MMA) [19,20]. Due to the collimator’s Rayleigh length, only a few millimeters in thickness is spared to accommodate the MOS shutter plate. Setting aside the volume for the package, only 1 mm or less in thickness is used for the actuation mechanism. For this reason, the electrostatic actuation has been chosen for shutter addressing rather than a scanned magnet [13,21]. Another option for MEMS shutter was a roll-blind mechanism [22,23] that curls up as an initial state due to the difference of thermal expansion coefficients of the layered materials. Such shutters could also be individually closed by the electrostatic addressing, but they usually require higher voltage at a cryostat temperature.

The shutter element in this work consists of a thin blade in a rectangular shape attached to a pair of supporting torsion bars. The shutter blade is suspended over an aperture hole such that the incoming light is blocked when the blade is kept in a horizontal position; when the shutter blade is retracted inward to the hole, on the other hand, the light is let through the slit. To open the shutter elements in arbitrary patterns, we have developed a cross-bar type addressing scheme that has been extended by using a third electrode integrated in a shutter element.

In this paper, we present the structures of the shutter elements and their arrayed format, and elaborate the electrostatic operation procedures that enable random access addressing. A MATLAB-Arduino controller has been developed to verify the addressing scheme. A shutter sub-cluster in a 3 × 3 format has been developed to demonstrate random addressing. This paper includes the extended results of the work reported in conference proceedings [17,18].

## 2. Multi-Object Spectrograph (MOS) Shutter Array

**Figure 2:**
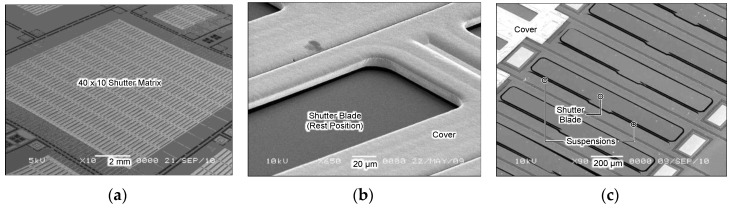
Scanning electron microscope (SEM) images of micro shutter array. (**a**) Entire view of a 40 × 10 array, (**b**) close-up view of the shutter blade and the cover plate, and (**c**) the shutter blade after removal of the cover plate.

Figure 2 shows the scanning electron microscope (SEM) images of the shutter array developed in this work [17]. A total of 8000 shutter elements is needed to cover the incoming collimated beam width of about two inches in diameter. Considering the processing yield of utilized silicon micromachining, it was thought to be difficult to implement such a large shutter array in a single wafer without any defects. Therefore, we chose a way to tile sub-cluster arrays in a 4 × 5 format to make up a shutter array system of a relatively large area.

The device shown in Figure 2a is a trial sub-cluster array that contains 40 × 10 shutter elements in a 20 × 20 mm area. Figure 2b is a close-up SEM view of the shutter blade; only a part of which is showing through an opening of 80 × 950 µm in area made in the cover plate. The full structures of the shutter blade are visible after removal of the cover plate, as shown in Figure 2c. The blade is 100 µm in length measured from the rotation center to the tip, and 1000 µm in width in the direction parallel to the torsion bars; each torsion bar is 450 µm long and 1.5 µm wide. The blade and the torsion bars are made of a silicon-on-insulator (SOI) layer of a uniform thickness of 1.4 µm. The cover plate is made of 12 µm thick electroplated nickel, which shows faint relief patterns of the underlying shutter structures.

**Figure 3:**
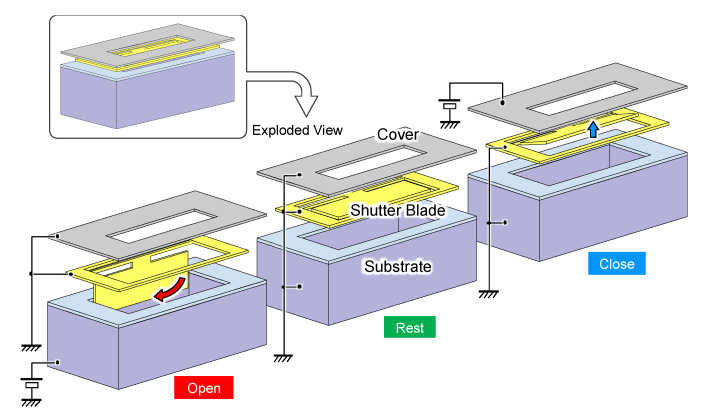
Exploded views of the electrostatic mechanism of micro shutter element. From left to right: open position (shutter blade retracted inward), rest (shutter blade at horizontal position), and close (shutter blade latched upward to the cover). The cover and the substrates are used to attract the shutter blade by the electrostatic force of the applied voltages.

The shutter blade is suspended over a through hole etched in the substrate, as illustrated in Figure 3. When no voltage is applied, the shutter blade remains flat at the rest position. Due to the metallic coating on the shutter, the incoming infrared light is rejected by the blade at this position but a fraction of light may scatter and leak through the tiny gaps around the blade. Therefore, the gap is firmly closed by electrostatically attracting the shutter blade to the cover using the voltage applied between the shutter and the cover. To let the incoming light through the slit, on the other hand, the shutter blade is retracted almost 90° inwards into the through hole by the electrostatic torque induced by another voltage applied to the bottom substrate; the surface of the through hole is coated with an electrical insulating silicon oxide, which prevents electrical short circuit upon contact with the shutter blade. A transparent substrate such as glass could be used as reported by Viereck et al. [24]. However, a silicon wafer with through holes was chosen in this work to minimize the transmissive loss through the aperture.

The fabrication process, which has been reported in detail elsewhere [17], is outlined as follows; a 1.4-µm-thick active layer of an SOI wafer was patterned by the deep reactive ion etching (DRIE) using reactive gases of SF_6_ and C_4_F_8_. The torsion springs were intended to have small elastic rigidity for the sake of low voltage operation, while the shutter blade was expected to be solid to keep its shape as flat as possible after metallic coating; after a few cycles of trial-and-error using the varieties of SOI wafers available in the market, we chose the 1.4-µm-thick SOI as a satisfactory thickness to design the torsion springs and the shutter blade. The cover structure was formed by two-step electroplating of metals, i.e., 10-µm-thick copper as a sacrificial layer and additional 12-µm-thick nickel as a structural layer. Two more layers were inserted between the plated metals: an adhesion promotor layer of 50-nm-thick chromium and a seed layer of 200-nm-thick gold. The combination of nickel and copper was chosen because of the large contrast of etching rate to keep the nickel structures intact after the selective sacrificial removal of copper without leaving any residue. Any metals of around 1 µm in thickness could be used to block the infrared and to conduct operation voltage; nonetheless, we used 12-µm-thick plated nickel because of the mechanical rigidity to keep the cover plate structures from collapsing under the electrostatic force. The copper thickness was chosen to design the drive voltage to be less than 10 V to close the blade. After selective removal of the plated copper, the gap was temporarily fixed by filling with Parylene-C formed by low pressure chemical vapor deposition (LPCVD). On the reverse side of the wafer, an aluminum layer was put as an etching mask for the through-hole process of the substrate by DRIE. The final sacrificial release was performed by removing the buried oxide by RIE process using CHF_3_. Finally, the temporal passivation layer Parylene-C was removed in an O_2_ plasma to release the shutter structures to be movable.

## 3. Electrostatic Actuation Models

**Figure 4:**
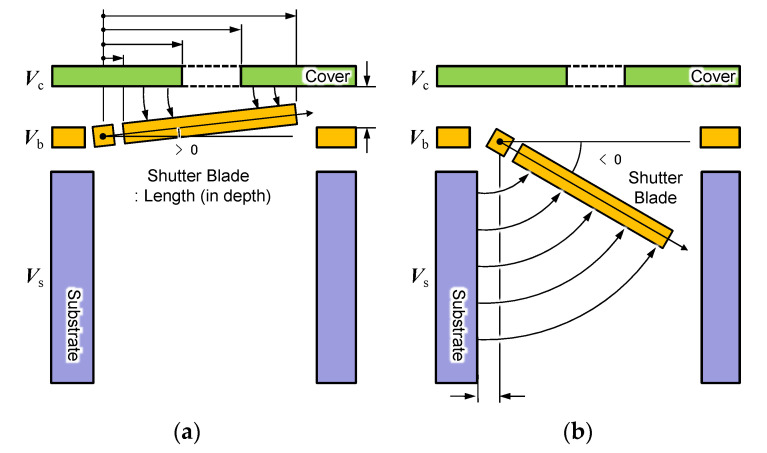
Side view of the three-port electrostatic mechanism. (**a**) Shutter blade being latched upwards to close the aperture, and (**b**) shutter blade being retracted inward to open the aperture. The electrostatic fields are represented by the arc for the simplicity of analysis. See Table 1 for the dimensions for the design parameters.

The electrostatic operation principle of the shutter blade is understood by using the analytical model shown in Figure 4. When the shutter blade is attracted upward to the cover plate, forming a positive angle θ with respect to the horizontal plane, we use the model shown in Figure 4a, where g is the initial gap between the horizontal plane of the substrate and the shutter blade [25]. Dimension a is the lateral distance to the blade’s inner edge, b and c are the positions of the aperture’s left- and right-hand side edges, respectively, and d is the distance to the blade’s tip, all measured from the rotation axis of the torsion bar. We set a moving axis x along the blade with its origin located at the torsion axis, and write the electrostatic force dF acting on a small section of length dx on the blade as

(1) $$dF=\frac{1}{2}\varepsilon_{0}\frac{W\;dx}{{\left(\;g-x\cdot \theta \;\right)}^{2}}V^{2},$$

where W is the blade’s width measured in the direction normal to the page plane, $\varepsilon_{0}\;\left(=8.854\times 10^{-12}\;F/m\right)$ is the dielectric constant of vacuum, and V is the electrical potential difference between the shutter blade and the cover plate. Owing to the geometrical condition that g ≪d, we presume that the elevation angle θ is a few degrees or less. Therefore, we utilize the formula for the electrostatic force of a parallel-plate actuator to represent the electrostatic behavior of the thin torsion plate, without considering the concentration of electrical fields on the blade edges. We also simplify the model by considering the electrostatic forces acting only in two zones on the blade, a≤x≤b and c≤x≤d, excluding the area corresponding to the aperture window in the cover plate. The electrostatic force in Equation (1) is converted into a net torque to close the shutter as

(2) $$T_{\mathrm{close}}=\frac{1}{2}\varepsilon_{0}\;W\;V^{2}\left\{\int_{a}^{b}\frac{x}{{\left(g-x\cdot \theta \right)}^{2}}dx+\int_{c}^{d}\frac{x}{{\left(g-x\cdot \theta \right)}^{2}}dx\right\}.$$

here, the integral term is expanded by using a variable transformation, g−x·θ=p, as

(3) $$\begin{array}{cc} I(a,b) & =\int_{a}^{b}\frac{x}{{\left(g-x\cdot \theta \right)}^{2}}dx\\ & =\int_{g-a\cdot \theta }^{g-b\cdot \theta }\frac{g-p}{\theta \;p^{2}}\left(-\frac{dp}{\theta }\right)\\ & =\frac{1}{\theta^{2}}\left\{g\left(\frac{1}{g-b\cdot \theta }-\frac{1}{g-a\cdot \theta }\right)-\ln\frac{g-a\cdot \theta }{g-b\cdot \theta }\right\}. \end{array}$$

Therefore, the net electrostatic torque in Equation (2) is rewritten as

(4) $$\begin{array}{cc} T_{\mathrm{close}}\left(\theta ,V\right) & =\frac{\varepsilon_{0}\;W\;V^{2}}{2}\left\{I\left(a,b\right)+I\left(c,d\right)\right\}\\ & =\frac{\varepsilon_{0}\;W\;V^{2}}{2\;\theta^{2}}\{g\left(\frac{1}{g-b\cdot \theta }-\frac{1}{g-a\cdot \theta }+\frac{1}{g-d\cdot \theta }-\frac{1}{g-c\cdot \theta }\right)-\ln\frac{\left(g-a\cdot \theta \right)\left(g-c\cdot \theta \right)}{\left(g-b\cdot \theta \right)\left(g-d\cdot \theta \right)}\} \end{array}$$

When the torsion blade is retracted into the through hole, on the other hand, we use another analytical model shown in Figure 4b. The actual device has a small lateral offset s between the substrate’s side wall and the torsion bar but this length is ignored for simplicity. In this model, we presume that the electrostatic flux profile is approximated by an arc with its center located at the torsion axis. The length of the arc that terminates on the shutter blade at position x is written as $x\;\left(\frac{\pi }{2}+\theta \right)$, where θ<0. Therefore, the electrostatic force dF acting on a small section length dx on the blade is [26]

(5) $$dF=\frac{1}{2}\varepsilon_{0}\frac{W\;dx}{{\left\{x\;\left(\frac{\pi }{2}+\theta \right)\right\}}^{2}}V^{2}.$$

In the same manner as for the electrostatic force induced by the cover plate, the electrostatic torque to attract the shutter blade to the side wall of the through hole is

(6) $$\begin{array}{cc} T_{\mathrm{substrate}}\left(\theta ,V\right) & =\frac{1}{2}\varepsilon_{0}\;W\;V^{2}\int_{a}^{d}\frac{x}{{\left\{x\;\left(\frac{\pi }{2}+\theta \right)\right\}}^{2}}dx\\ & =\frac{\varepsilon_{0}\;W\;V^{2}}{2{\left(\frac{\pi }{2}+\theta \right)}^{2}}\;\ln\frac{d}{a}. \end{array}$$

The electric field concentration on the shutter’s edge is ignored.

We use the linear model for the elastic rigidity of the suspension that is

(7) $$T_{\mathrm{torsion}}\left(\theta \right)=k\;\theta .$$

Parameter k is the elastic rigidity described as [27]

(8) $$k=2\times \frac{G\;w_{s}\;t_{s}{}^{3}}{3\;l_{s}}\left(1-\frac{192}{\pi^{5}}\;\tanh\frac{\pi \;w_{s}}{2t_{s}}\;\right),$$

where $w_{s}$, $l_{s}$*,* and $t_{s}$ are respectively the width, the length, and the height of a torsion spring of a rectangular cross section ($t_{s}\leq w_{s}$*),* and G (= 63.1 GPa) is the shear modulus of rigidity of silicon. The rigidity has been doubled because of the two torsion bars attached to the shutter blade. The mechanical resonant frequency of the shutter is modelled by

(9) $$f_{0}=\frac{1}{2\;\pi }\sqrt{\frac{k}{I_{m}}},$$

where $I_{m}$ is the moment of inertia of the suspended shutter blade that is calculated by

(10) $$I_{m}=\rho \int_{a}^{d}W\;t\;x\;dx,$$

where t is the thickness of the blade, which is equal to the suspension height $t_{s}$ in the developed device, and ρ ($2.33\times 10^{3}$ kg/m^3^) is the density of silicon.

In an actual device, judging from the reflection observed under the optical microscope, the shutter blade at the closed position was found to make a first contact with the bottom of the cover plate at its tip, and then the entire blade was pulled upwards to firmly close the gap with increasing voltage. At this position, the short circuit was avoided because of the thin silicon oxide film remaining on the shutter blade. Optical microscope observation performed after the removal of the cover plate also revealed that the shutter blade deflected laterally towards the sidewall of the substrate due to the electrostatic force. Details of the blade’s three-dimensional motion should be handled by numerical computation such as boundary element method; therefore, the simplified analytical models discussed here should be used to predict the electrostatic pull-in voltage that takes place before causing such an excess mechanical deformation of the shutter blade.

We used the electrostatic torque expression in Equation (4) or (6) to be equated with the mechanical restoring torque in Equation (7) to numerically calculate the blade angle θ as a function of the applied voltage V. An equivalent circuit model was developed as a mechano-electric module in the electrical circuit simulator LTspice^TM^; the methodology to convert the analytical model into an equivalent circuit had been reported elsewhere [28]. Parameters used for the simulation are listed in Table 1.

**Figure 5:**
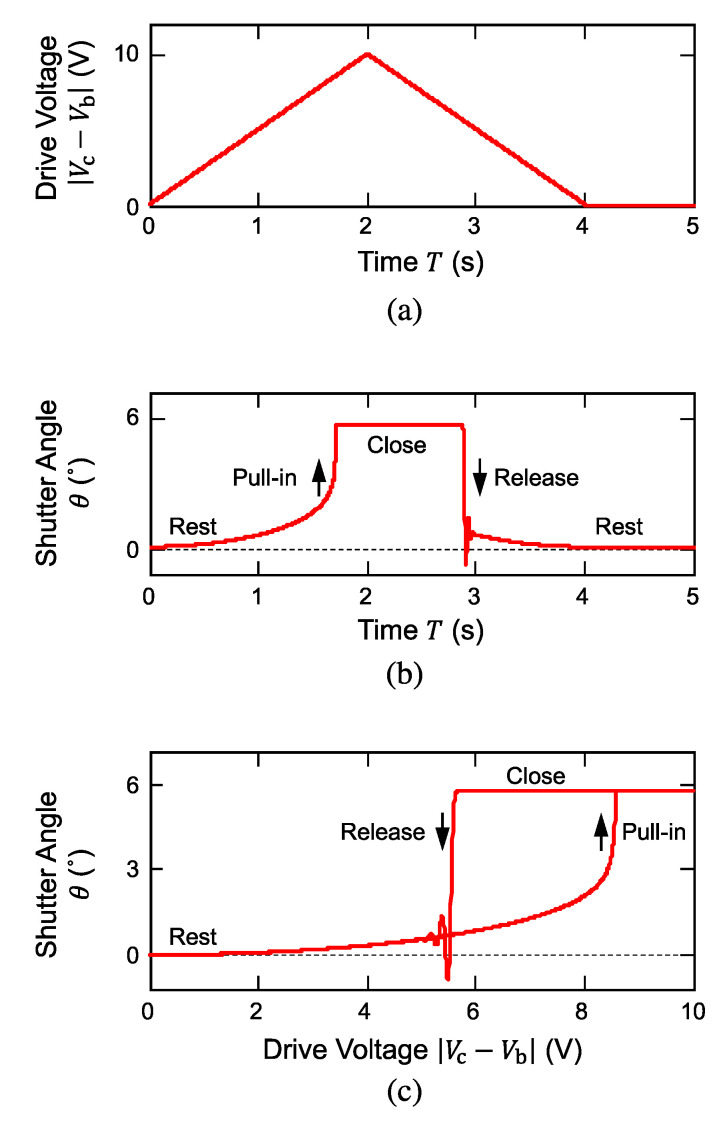
Equivalent-circuit simulation results of electrostatic shutter operation (rest-close). (**a**) Drive voltage waveform used for simulation and (**b**) calculated shutter angle waveform. (**c**) Alternative presentation of the shutter angle as a function of drive voltage. Electrostatic hysteresis loop has been reproduced by simulation.

A monopolar triangular wave of 10 V max shown in Figure 5a was used as a differential voltage, which was a potential difference between the voltage applied to the cover plate, $V_{c}$, and that to the shutter blade, $V_{b}$, and the transient behavior of the blade angle θ was observed. The slew rate of the ramped voltage was set to be intentionally slow (~2 s) compared with the time constant (~37 ms) corresponding to the mechanical resonance (~27 Hz) of the shutter blade so that the calculated transient behavior could be regarded as quasi-static characteristics.

When the drive voltage was increased, the shutter angle gradually increased and finally tripped to the mechanical limiter at a voltage of around 8.5 V as shown in Figure 5b; this sequence is a typical electrostatic pull-in behavior of a semi-parallel plate actuator. At this voltage and higher, the electrostatic attractive torque became greater than the mechanical restoring force, and the blade was brought into contact with the cover plate. The contact was maintained so long as the drive voltage was kept higher than a certain voltage of around 5.5 V, which is usually referred to as the release voltage, where the blade was released from the pull-in contact. The release was followed by a damped oscillation at a frequency that corresponded to the electromechanical resonance of the suspended shutter blade.

Using the time sequences of the drive voltage and the shutter angle respectively shown in Figure 5a,b, we replot the angle as a function of applied voltage as shown in Figure 5c. Owing to the pull-in and release behaviors that took place at different voltages, the operation curve had a large hysteresis loop. Taking advantage of this behavior, the voltage to hold the blade at the close position could be lowered once the pull-in occurred. This behavior is beneficial in designing the control system of the shutter array all in a passive manner as discussed later.

**Figure 6:**
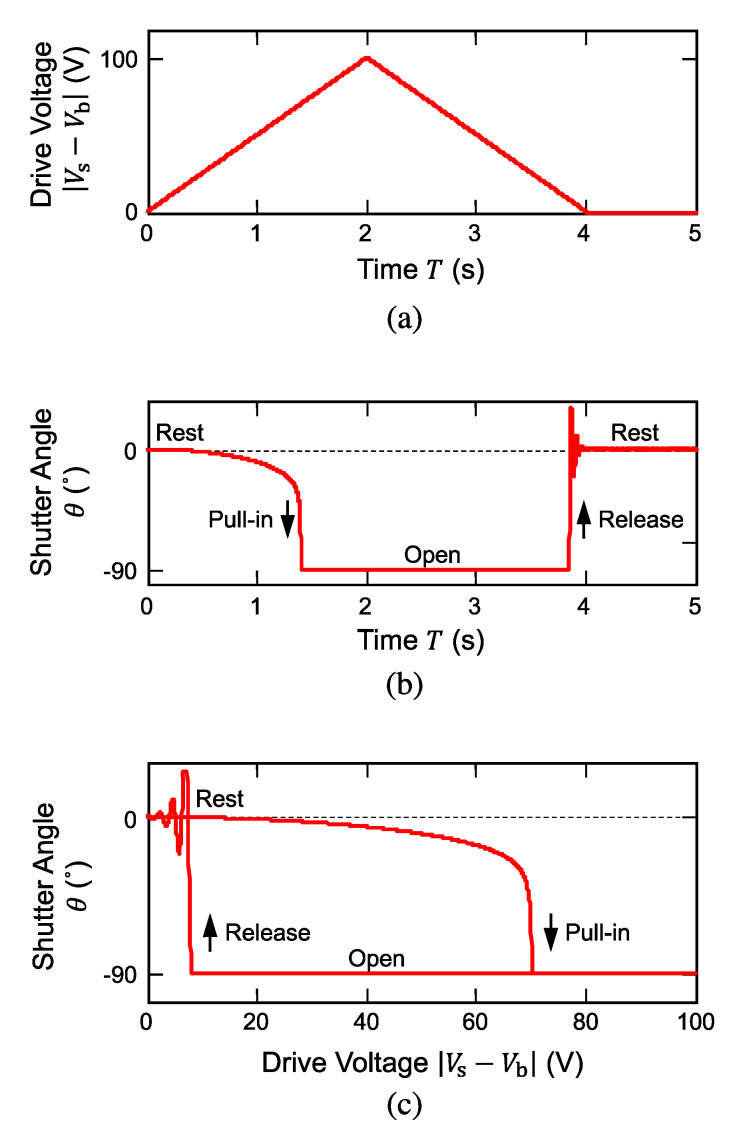
Equivalent-circuit simulation results of electrostatic shutter operation (rest - open). (**a**) Drive voltage waveform used for simulation and (**b**) calculated shutter angle waveform. (**c**) Shutter angle as a function of drive voltage.

Electrostatic simulation was also performed for the shutter-open sequence as shown in Figure 6. Once again, the angle is plot as a function of voltage $\left|V_{s}-V_{b}\right|$, which is a differential voltage applied between the substrate and the shutter blade. Owing to the large initial angular gap (90°) in the geometrical configuration, a relatively high voltage of 78 V was needed as shown in Figure 6a to allow the electrostatic pull-in opening. Similar to the close operation, the shutter blade exhibited electrostatic pull-in and release behaviors as shown in Figure 6b, which was also converted into the angle-voltage plot shown in Figure 6c. Because of the large contrast of the blade angles between the open and the rest positions, the hysteresis curve had a wide opening so that the release of the blade did not occur until the voltage was lowered to 8 V or less; this characteristic is also beneficial for electrostatic operation in a sense that the drive voltage could be lowered to a small value, thereby reducing the risk of electrical breakdown of the insulating materials.

**Figure 7:**
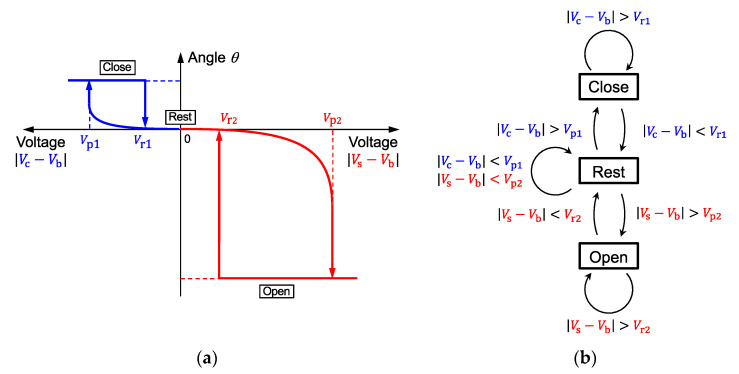
Simplified behavior model of electrostatic shutter operation (close, rest, and open without considering the damped oscillation). (**a**) Unified plot of the shutter angle as a function of applied voltage and (**b**) state transition diagram using the voltage combination as a trigger for transition.

The hysteresis loops shown in Figure 5c and Figure 6c are merged in a simplified diagram as shown in Figure 7a; the dynamic behavior of damped oscillation is omitted. The vertical axis is the blade’s angle, while the lateral axes are the drive voltages; the left-pointing axis is the potential difference between the cover plate ($V_{c}$) and the shutter blade ($V_{b}$), and that on the right-hand side is the voltage between the substrate ($V_{s}$) and the shutter blade ($V_{b}$). By using the combinations of the differential voltages, the shutter blade position can be set at either the rest, close, or open position. Note that the angle is governed not solely by the electrical potentials of the individual electrodes but the differential voltages $\left|V_{c}-V_{b}\right|$ and $\left|V_{s}-V_{b}\right|$ will determine the shutter status. Characteristic voltages $V_{p1}$ and $V_{r1}$ are the pull-in and the release voltages for the shutter’s closing motion, respectively, and $V_{p2}$ and $V_{r2}$ are those for the shutter’s opening.

Provided that the shutters are firmly latched to the close state with a voltage higher than the release voltage ($V_{r1}$), no direct transition would occur from the close state to the open state but it always needs to go to the rest position by removing the differential voltage. In other words, the dimensions of the shutter device could be designed to latch the blade firmly at the closed position no matter what voltage is applied to the substrate.

The shutter status on the hysteresis loop shown in Figure 7a is simplified to a state transition diagram in Figure 7b. Starting from the rest position by fulfilling the conditions that $\left|V_{c}-V_{b}\right|<V_{p1}$ and $\left|V_{s}-V_{b}\right|<V_{p2}$, the shutter could be pulled into the close position by altering the condition for $V_{c}$ and $V_{b}$ to $\left|V_{c}-V_{b}\right|>V_{p1}$. Once the blade is latched to the close position, the status remains so long as the voltage condition $\left|V_{c}-V_{b}\right|>V_{r1}$ is maintained, regardless the value of $V_{s}$. To open the shutter, it should be released to the rest position first by changing the $V_{c}$ and $V_{b}$ conditions to $\left|V_{c}-V_{b}\right|<V_{r1}$, and then the blade should be attracted to the substrate’s side wall by the voltages that fulfill $\left|V_{s}-V_{b}\right|>V_{p2}$. Once this open condition is made, the shutter is not affected by $V_{c}$ but it only responds to $\left|V_{s}-V_{b}\right|<V_{r2}$ when it returns to the rest position.

## 4. Passive Addressing of Shutter Array

**Figure 8:**
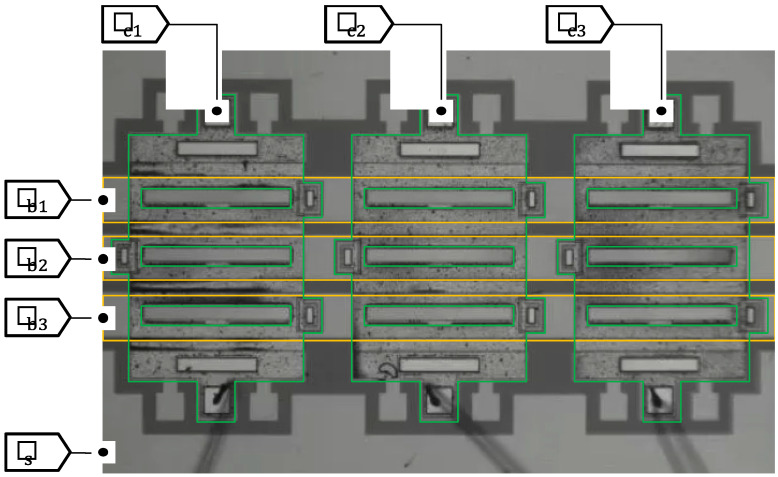
Electrical interconnection of the 3×3 micro shutter array. The shutter blades are connected in the lateral direction to form three rows, while the cover plates form three columns. The substrate is used as a common electrode.

A shutter array was developed in a 3×3 format as shown in Figure 8, which was used to verify the all-passive addressing scheme similar to the cross-bar operation. The shutter blades made of a single SOI layer were electrically connected in the lateral direction to form three rows of electrodes $V_{b1}$, $V_{b2}$, and $V_{b3}$, while the cover plates made of nickel were connected in the orthogonal direction to make three columns of electrodes $V_{c1}$, $V_{c2}$, and $V_{c3}$. The substrate was used as a common electrode $V_{s}$ for the shutter elements. These seven electrodes were electrically isolated from each other.

**Figure 9:**
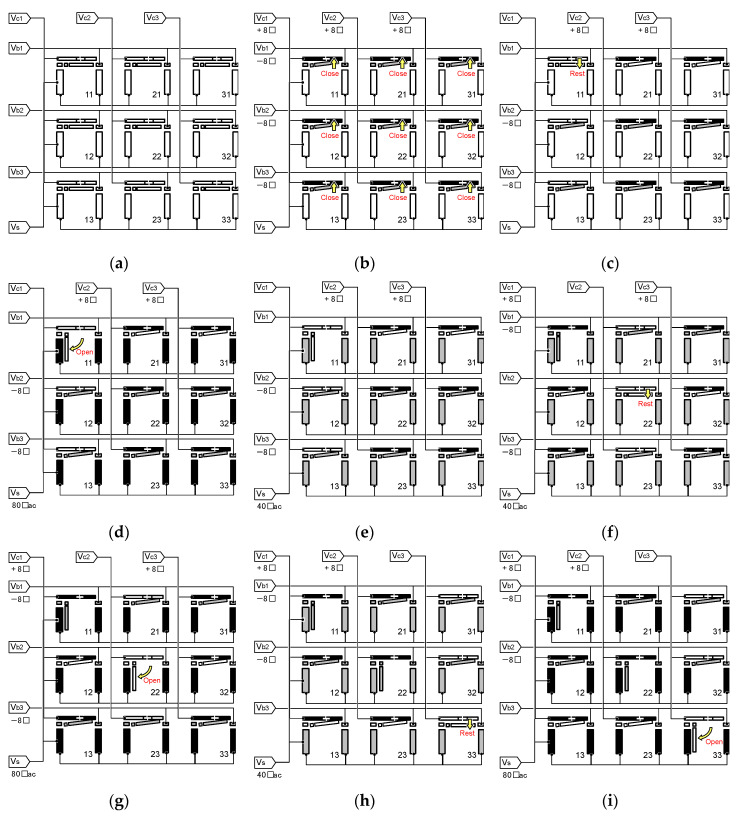
Operation sequence to open an arbitrary one of the shutter elements. Side views of the shutter, the cover plate, and the substrate are shown. (**a**) Initial state, (**b**) all closed, (**c**) element-11 released, (**d**) element-11 opened, (**e**) hold, (**f**) elemnt-22 released, (**g**) element-22 opened, (**h**) element-33 released, and (**i**) element-33 opened.

Sequential addressing any one of the 3×3 shutter elements is possible with these seven electrodes only, without affecting the unintended shutter elements. For instance, a voltage sequence to open the diagonal three elements is schematically shown in Figure 9, in which the side views of the shutters, the cover plates, and the substrate are shown. In this sequence, we used 0 V or +8 V_dc_ for $V_{c1}$, $V_{c2}$, and $V_{c3}$, while 0 V or −8 V_dc_ for $V_{b1}$, $V_{b2}$, and $V_{b3}$. The substrate voltage is either 80 V_ac_ to open or 40 V_ac_ to hold the shutter. Square wave AC voltages at 10 Hz without any biasing offset are used to avoid permanent stiction of the shutter blades to the substrate by electrostatic charge-up. Owing to the short transition time of the square-wave voltage, usually being less than 10 µs, which is negligible compared with the time constant of the shutter blade’s response, the blade remains at the pull-in position even though the operation frequency (10 Hz) is lower than the shutter’s resonance (~27 Hz).

When all the electrodes are electrically grounded, the shutter elements remain at the rest position as schematically shown in Figure 9a. When voltages of the opposite polarities are applied to all the cover plates (+8 V) and the shutters (−8 V), the differential voltages exceed the pull-in voltage, $V_{p1}$, and all the shutter elements are closed as shown in Figure 9b. To open the shutter element located at position 11, for instance, the voltage applied to the cover in the first column ($V_{c1}$) and that to the shutter blade in the first row ($V_{b1}$) are removed to free the shutter blade to the rest position, as shown in Figure 9c; the other shutter blades in the same column or row are still held at the close position because of the remaining differential voltages which are greater than the release voltage $V_{r1}$. Once the shutter is selectively released, it can be attracted to the open position by the voltage applied to the substrate, 80 V_ac_, which is greater than $V_{p2}$, as shown in Figure 9d, without affecting other shutters at the closed position. Opening the shutter requires a rather high voltage but momentarily for 100 ms only, after which the voltage can be reduced to 40 V_ac_, as shown in Figure 9e to lower the risk of electrical breakdown; with this voltage being greater than $V_{r2}$, the shutter blade is held at the open position. To open the next shutter at position 22, the same sequence is repeated to selectively release the shutter to the rest position as shown in Figure 9f and then open it by applying a high voltage to the substrate as shown in Figure 9g. Subsequently, the shutters at position 33 are opened through steps shown in Figure 9h,i. The sequence can be repeated unlimitedly to open any one of the remaining shutters. In addition, this scheme is scalable to M×N arrays by using M+N+1 addressing electrodes, including the substrate, rather than M×N+1 individual electrodes.

Operating an array of electrostatic actuators is a classic problem, and many preceding studies have been reported. For instance, Braun et al. used an orthogonal set of electrodes to perform a cross-bar operation for an array of two-port electrostatic actuators [29]. They utilized the hysteresis loop of electrostatic actuation and sequentially zipped in an actuator element. They also performed a statistical study to demonstrate an operational reliability of more than 99.9% for 20×20 elements. The same scheme is theoretically applicable to this work, by sequentially choosing the shutter elements at the closed position and simultaneously opening the rest of the shutters at the end. However, the pull-in voltage for the close position, $V_{p1}$, in this work was not uniform in the arrayed shutters due to the distributed gap length between the shutter blades and the overlying cover plate; simultaneous opening was thus not possible, and the shutter selection sequence frequently failed to leave other shutters at the close positions. Instead, we used an alternative method to sequentially release and open a shutter element, by taking the advantage of bi-directional electrostatic operation, which was made possible with the three-port configuration of the shutter structures.

Use of a built-in electrical switching circuit is a straightforward method to achieve random access of electrical element as summarized in [30]. However, we have chosen all passive electrical access to avoid thermal radiation from the integrated circuits. Another idea is to use segmented electrodes on the substrate side to perform electrostatic cross-bar operation to open the shutter. However, making isolated electrode patterns on the side walls of the substrate was found to be difficult to maintain a high processing yield. Therefore, we used the whole piece of the substrate as a common electrode to open the shutters.

## 5. Demonstration

**Figure 10:**
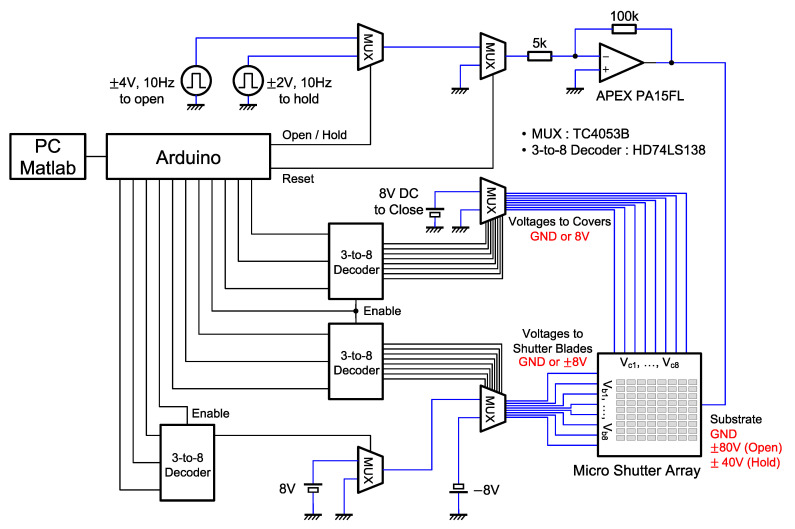
Block diagram of electrical circuit for addressing shutter elements. The digital output from the Arduino are used to control the pairs of decode and multiplexer (MUX) that deliver analog drive voltages to the rows and columns of the MEMS micro shutter array to individually close the shutter blades. High-voltage operation is applied to the common substrate that attracts the shutter blades to the open position.

A MATLAB^®^-based control system was developed to demonstrate all passive addressing of the shutter matrix. Figure 10 shows the block diagram of the controller. The digital outputs of Arduino microprocessor (Arduino Uno SMD R3, Arduino, LLC, Boston, MA, US) were used to control three pieces of 3-to-8 decoders (HD74LS138, Renesas Electronics Corp., Tokyo, Japan); each decoder transmits eight bits of commanding signals to the subsequent multiplexers (MUX, TC4053B, Toshiba Corp., Tokyo, Japan), which had eight switches in parallel to choose one of two input lines each. The combination of these decoders and multiplexers gives voltages to the shutter covers ($V_{c1},\;V_{c2},\;\cdots ,\;V_{c8}$) and the shutter blades ($V_{b1},\;V_{b2},\;\cdots ,\;V_{b8}$). The output voltage could be chosen from 0 V, +8 V, and −8 V. On the other hand, the drive voltage to the substrate was generated from the external function generators (±2 V and ±4 V at 10 Hz), one of which was chosen by the multiplexer and amplified in the analog voltage booster (PA15FL, APEX Microtechnology, Tucson, AZ, US) to produce square waves of either ±40 V or ±80 V.

The developed controller was capable of handling an 8×8 matrix at a time but we used a 3×3 subset to verify the scheme of control sequence. Typical Arduino has 19 digital output ports; sparing two outputs for the selectors in the substrate voltage line (open/hold and reset) and extra two outputs for the enable lines, we would use the remaining 15 outputs to control the decoders. For instance, the 40×10 shutter array shown in Figure 2a is addressable by using 6 bits for 64 rows of shutter blades and 4 bits for 16 columns of cover plates, making up a total addressing capacity of $2^{6}\times 2^{4}$ elements, which can be implemented using a typical Arduino architecture.

**Figure 11:**
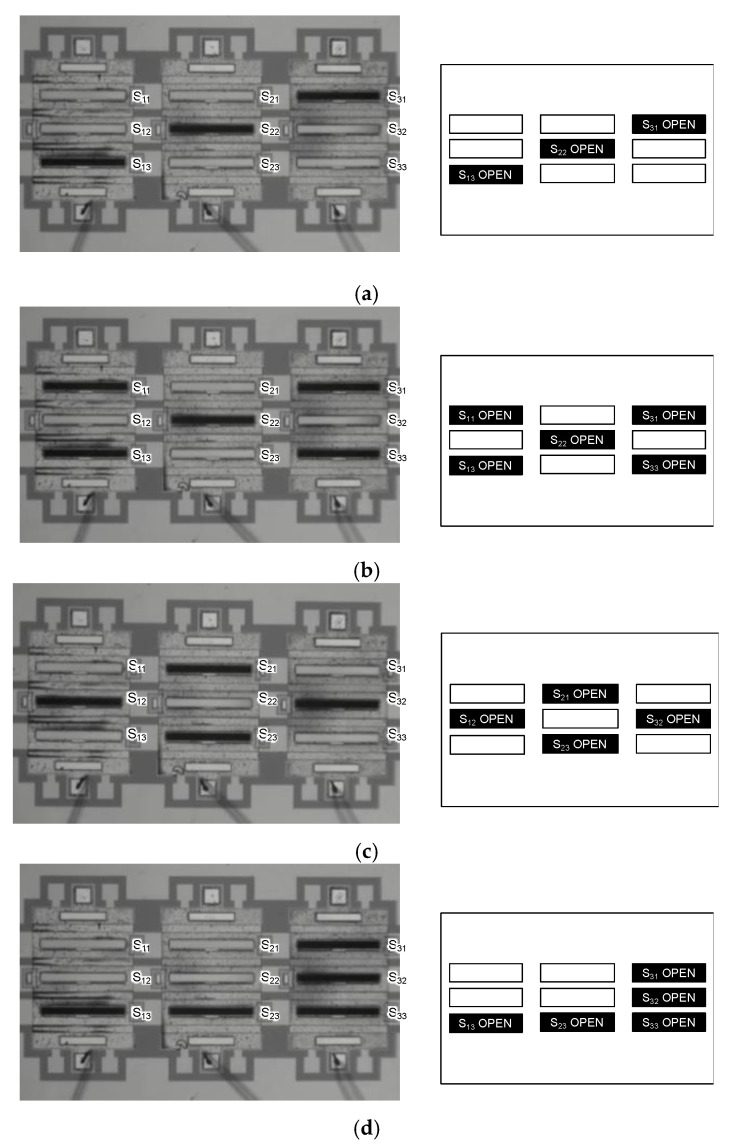
Examples of 3×3 shutter array operation. (**a**) Diagonal three elements ($S_{13}$, $\;S_{22}$ and $\;S_{31}$ ) are addressed. Shutters are also addressed in (**b**) a cross shape ($S_{11}$, $\;S_{13}$, $S_{31}$, $S_{33}$ and $\;S_{22}$ ), (**c**) a diamond ($S_{12}$, $S_{23}$, $S_{32}$ and $\;S_{21}$ ), and (**d**) a mirror-flipped L-shape ($S_{13}$, $S_{23}$, $S_{33}$, $S_{32}$ and $\;S_{31}$ ).

Figure 11 shows the results of sequential addressing test of the 3×3 shutter matrix. The shutter blade at the rest or close position reflects the illuminating light of the microscope, and therefore it looks bright in the photograph. The black slots are the shutters that have been electrically addressed. In Figure 11a, only one pixel out of every column was chosen, thereby to form a diagonal line at positions 13, 22, and 31. It was possible to choose more than two shutter elements in a single column or row to form cross-out and diamond shapes as respectively shown in Figure 11b,c. Addressing all the three elements in a column and a row was also possible as shown in Figure 11d. From these results, the developed control system and the sequential addressing approach were capable of addressing arbitrary ON/OFF patterns to meet the requirement for the reconfigurable slits array for multi-object spectroscopy.

## 6. Conclusions

Different from the addressing principles of micro shutter array (MSA) devices for galactic astronomy that used a combination of electrostatic latching and scanned magnet actuation, we newly developed an all electrostatic random addressing scheme by using three-port configuration of a micro shutter element that had the shutter blade, cover plate, and substrate as electrodes for electrostatic actuation. An analytical model for the three-port electrostatic actuation was developed to predict the behavior of shutter blade controlled by the sequence of voltage application to individual electrodes. A systematic voltage sequence was found to address any one of the M×N element to configure arbitrary open/close patterns of matrix, which was verified by the experiment using a MATLAB-coded electronic controller. The electrostatic operation mechanism was integrated within a thin substrate of less than 1 mm, which could be accommodated in a Rayleigh length about the focal plane of the spectroscope optics. An extended version of this addressing operation is under development for telemetric operation of a ground-based infrared observatory.

## Figures and Tables

**Table 1:** Design parameters used in electrostatic simulation

Element	Parameter	Symbol	Value	Unit
Shutter Blade	Width (parallel with torsion bars)	W	1000	µm
	From Torsion Bar to Inner Edge	a	10	µm
	From Torsion Bar to Tip	d	100	µm
	Thickness (= SOI Thickness)	t	1.4	µm
Cover Slit	Torsion Bar to Left Edge	b	20	µm
	Torsion Bar to Right Edge	c	90	µm
	Air Gap	g	10	µm
Suspension	Width	$w_{s}$	1.5	µm
	Length	$l_{s}$	450	µm
	Height (= SOI Thickness)	$t_{s}$	1.4	µm
	Lateral Offset from Side Wall	s	10	µm
Silicon	Shear Modulus of Rigidity	G	63.1	GPa
	Density	ρ	$2.23\times 10^{3}$	kg/m^3^
